# The genome sequence of *Aesculus hippocastanum* L., 1753 (Sapindales: Sapindaceae)

**DOI:** 10.12688/wellcomeopenres.27204.1

**Published:** 2026-08-31

**Authors:** Markus Ruhsam, Peter M. Hollingsworth, Royal Botanic Garden Edinburgh Genome Acquisition Lab, Plant Genome Sizing Collective, Darwin Tree of Life Barcoding collective, Wellcome Sanger Institute Tree of Life Management, Samples and Laboratory team, Wellcome Sanger Institute Scientific Operations: Sequencing Operations, Wellcome Sanger Institute Tree of Life Core Informatics team, Tree of Life Core Informatics collective, Darwin Tree of Life Consortium

**Affiliations:** 1 Royal Botanic Garden Edinburgh, Edinburgh, Scotland, UK

**Keywords:** Aesculus hippocastanum, Horse-chestnut tree, genome sequence, chromosomal, Sapindales

## Abstract

We present a genome assembly of *Aesculus hippocastanum* (Horse-chestnut tree; Streptophyta; Magnoliopsida; Sapindales; Sapindaceae). The assembly consists of two haplotypes with total lengths of 513.59 megabases and 506.90 megabases. Most of haplotype 1 (98.22%) is scaffolded into 20 chromosomal pseudomolecules. Haplotype 2 was assembled to scaffold level. The mitochondrial sequence has a length of 507.21 kilobases and the plastid genome assembly has a length of 156.08 kilobases. This assembly was generated as part of the Darwin Tree of Life project, which produces reference genomes for eukaryotic species found in Britain and Ireland.

## Species taxonomy

Eukaryota; Viridiplantae; Streptophyta; Streptophytina; Embryophyta; Tracheophyta; Euphyllophyta; Spermatophyta; Magnoliopsida; Mesangiospermae; eudicotyledons; Gunneridae; Pentapetalae; rosids; malvids; Sapindales; Sapindaceae; Hippocastanoideae; Hippocastaneae; *Aesculus*; *Aesculus hippocastanum* L., 1753 (NCBI:txid43364).

## Background

*Aesculus hippocastanum* L. is a deciduous broadleaved tree native to the mountainous regions of the Balkan Peninsula, where it persists as a relict in small, isolated populations in Albania, Greece, North Macedonia and a single disjunct locality in Bulgaria ( Tzonev *et al.*, 2024; Walas *et al.*, 2019). In its native range, the species is largely confined to humid mountain ravines, stream valleys and mixed mesophytic forests ( Tzonev *et al.*, 2024), however Horse chestnut is now widely cultivated throughout temperate regions of the world as an ornamental tree. It is generally considered a light-demanding to moderately shade-tolerant species that can establish beneath forest canopies but typically achieves its greatest growth and reproductive output in canopy openings, woodland edges and other well-lit habitats ( Tzonev *et al.*, 2024). Natural populations are frequently associated with refugial forest environments that buffer climatic extremes and maintain high humidity. Genetic studies indicate that despite the fragmented distribution of the species, many populations retain substantial levels of genetic diversity, reflecting persistence in multiple glacial refugia across the Balkans ( Walas *et al.*, 2019).

*Aesculus hippocastanum* reproduces sexually through large upright panicles of insect-pollinated flowers and produces large seeds (“conkers”) that are dispersed primarily by gravity, with occasional secondary dispersal by water in riparian habitats ( Walas *et al.*, 2019). The species exhibits a complex breeding system and is andromonoecious, producing both male and hermaphroditic flowers within the same inflorescence, with approximately 73% of flowers functioning as male ( Weryszko-Chmielewska & Chwil, 2017). Hermaphroditic flowers are protogynous, with the female phase preceding pollen release, thereby reducing self-fertilisation and favouring cross-pollination ( Tegrovsky & Pany, 2019). Flowering progresses sequentially through the inflorescence, prolonging floral display and pollinator attraction. Individual flowers exhibit a remarkable colour change during anthesis whereby the yellow nectar guides at the base of the petals turn orange-red following pollination. Experimental studies have demonstrated that pollinated flowers change colour more rapidly than unpollinated flowers, indicating that the colour shift functions as an honest signal of floral reward status ( Tegrovsky & Pany, 2019). Because bees readily perceive yellow but have limited sensitivity to red wavelengths, this transition directs pollinators towards newly opened, nectar-rich flowers and enhances pollination efficiency ( Tegrovsky & Pany, 2019).

Horse chestnut contains a diverse array of secondary metabolites distributed across seeds, bark, and leaves, with strong variation in chemical composition between tissues. The seeds are particularly rich in triterpenoid saponins collectively known as escin, which constitute the major bioactive constituents of *Aesculus hippocastanum* and are responsible for many of its pharmacological properties ( Idris *et al.*, 2020). In addition to saponins, seeds also contain flavonoids, including quercetin and kaempferol glycosides, which contribute to antioxidant activity ( Idris *et al.*, 2020; Kapusta *et al.*, 2007). The bark is characterised by coumarins such as esculin and fraxin, as well as phenolic compounds including procyanidins and other tannin-like substances, which are associated with defensive and antioxidant functions in plant tissues ( Owczarek *et al.*, 2021). Together, these compounds form a chemically diverse defence system that contributes to resistance against herbivores and pathogens and underpins the species’ well-known medicinal applications in human vascular disorders ( Idris *et al.*, 2020; Owczarek *et al.*, 2021).

The species supports a range of associated insects and microorganisms and has become particularly notable as the primary host of the horse-chestnut leaf-miner ( *Cameraria ohridella*), an invasive moth whose larvae can cause severe leaf damage and premature browning of foliage in trees across Europe ( Freise *et al.*, 2002; Thalmann *et al.*, 2003). The tree is also susceptible to diseases such as bleeding canker caused by *Pseudomonas syringae* pv. *aesculi*, which has become an important emergent pathogen of horse chestnut in Europe ( Green *et al.*, 2009).

The chromosome number of *Aesculus hippocastanum* is 2 *n* = 40, a number that appears to be conserved throughout the genus *Aesculus,* indicating a base chromosome number of *x* = 20 ( Krahulcová *et al.*, 2017).

**Figure 1.**
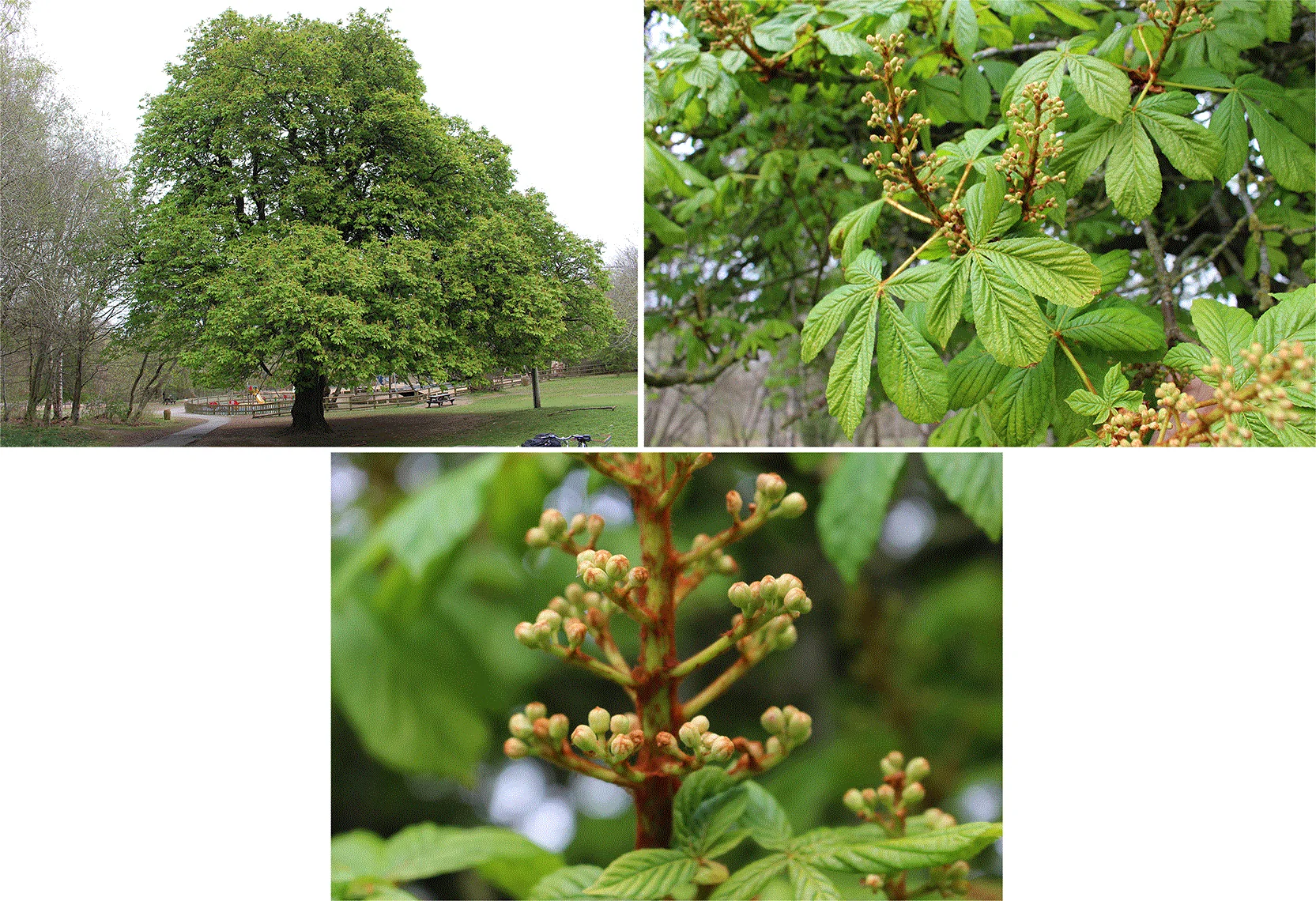
Photographs of the *Aesculus hippocastanum* (drAesHipp1) specimen from which samples were taken for genome sequencing.

We present a chromosome-level genome sequence for *Aesculus hippocastanum,* the Horse-chestnut. The assembly was produced using the Tree of Life pipeline from a specimen collected in Vogrie Country Park, UK ( Figure 1). It was generated as part of the Darwin Tree of Life Project, which aims to generate high-quality reference genomes for all named eukaryotic species in Britain and Ireland to support research, conservation, and the sustainable use of biodiversity ( Darwin Tree of Life Project Consortium, 2022).

## Methods

### Sample acquisition, flow cytometry and DNA barcoding

A specimen of *Aesculus hippocastanum* (specimen ID EDTOL01199, ToLID drAesHipp1; Figure 1) was used for genome sequencing. Leaves were collected from Vogrie Country Park, UK (latitude 55.8588, longitude −2.991) on 2021-04-27. The samples were collected and identified by Markus Ruhsam (Royal Botanic Garden Edinburgh). The herbarium voucher associated with the sequenced plant is E01358509 and is deposited in the herbarium of RBG Edinburgh (E) https://data.rbge.org.uk/herb/E01358509.

The genome size was estimated by flow cytometry following the ‘one-step’ method outlined in Pellicer *et al*. (2021) and using propidium iodide as the fluorochrome. The General Purpose Buffer (GPB) supplemented with 3% PVP and 0.08% (v/v) beta-mercaptoethanol was used for isolation of nuclei ( Loureiro *et al.*, 2007), and the internal calibration standard was *Petroselinum crispum* ‘Champion Moss Curled’ with an assumed 1C-value of 2 200 Mb ( Obermayer *et al.*, 2002).

The initial identification was verified by an additional DNA barcoding process according to the framework developed by Twyford *et al*. (2024). Part of the plant specimen was preserved in silica gel desiccant ( Chase & Hills, 1991). DNA extracted from the dried plant was amplified by PCR for standard barcode markers, with the amplicons sequenced and compared to public sequence databases including GenBank and the Barcode of Life Database (BOLD) ( Ratnasingham & Hebert, 2007). Following whole genome sequence generation, the relevant DNA barcode region was also used alongside the initial barcoding data for sample tracking at the WSI ( Twyford *et al.*, 2024). The standard operating procedures for Darwin Tree of Life barcoding are available on protocols.io.

### Nucleic acid extraction

Protocols for high molecular weight (HMW) DNA extraction developed at the Wellcome Sanger Institute (WSI) Tree of Life Core Laboratory are available on protocols.io ( Howard *et al.*, 2025). The drAesHipp1 sample was weighed and triaged to determine the appropriate extraction protocol. For HMW DNA extraction, 65.000 mg of leaf tissue was used. Tissue was homogenised by cryogenic bead beating. HMW DNA was extracted using sorbitol washing followed by the Plant Organic Extraction protocol. DNA was sheared into an average fragment size of 12–20 kb following the Megaruptor®3 for LI PacBio protocol. Sheared DNA was purified by automated SPRI (solid-phase reversible immobilisation), using AMPure PB beads (Pacific Biosciences) and the Thermo Fisher KingFisher™ Apex to eliminate shorter fragments and concentrate the DNA. The concentration of the sheared and purified DNA was assessed using a Nanodrop spectrophotometer and Qubit Fluorometer using the Qubit dsDNA High Sensitivity Assay kit. Fragment size distribution was evaluated by running the sample on the FemtoPulse system. For this sample, the final post-shearing DNA had a Qubit concentration of 19.9 ng/μL and a yield of ng, with a fragment size of 20.0 kb. The 260/280 spectrophotometric ratio was 1.92, and the 260/230 ratio was 2.25.

### PacBio HiFi library preparation and sequencing

Library preparation and sequencing were performed at the WSI Scientific Operations core. Libraries were prepared using the SMRTbell Prep Kit 3.0 (Pacific Biosciences) according to the manufacturer’s instructions. The kit includes reagents for end repair/A-tailing, adapter ligation, post-ligation SMRTbell bead clean-up, and nuclease treatment. Size selection and clean-up were performed using diluted AMPure PB beads (Pacific Biosciences). DNA concentration was quantified using a Qubit Fluorometer v4.0 (ThermoFisher Scientific) and the Qubit 1X dsDNA HS assay kit. Final library fragment size was assessed with the Agilent Femto Pulse Automated Pulsed Field CE Instrument (Agilent Technologies) using the gDNA 55 kb BAC analysis kit.

The sample was sequenced on a Revio instrument (Pacific Biosciences, California, USA). Prepared libraries selected for multiplexing were pooled based on genome size, library molarity, and the intended plex level. Primers were annealed and polymerases were bound to generate circularised complexes, following the manufacturer’s instructions. Complexes were purified using SMRTbell beads, diluted to the Revio loading concentration, and spiked with a Revio sequencing internal control. The pooled libraries were sequenced on a Revio 25 M Plex SMRT cell in a 2-plex run. SMRT Link software (Pacific Biosciences), a web-based workflow manager, was used to configure and monitor the run and to carry out primary and secondary data analysis.

### Hi-C

***Sample preparation and crosslinking***

Hi-C data were generated from the leaf tissue of drAesHipp1 using the Arima-HiC v2 kit (Arima Genomics). Tissue was finely ground using the Covaris cryoPREP Dry Pulverizer (Covaris), and then subjected to nuclei isolation. Nuclei were isolated using a modified protocol based on the Qiagen QProteome Cell Compartment Kit (Qiagen), in which only the Lysis and CE2 buffers were used, with QIAshredder spin columns. After isolation, nuclei were fixed using formaldehyde to a final concentration of 2% to crosslink the DNA. The crosslinked DNA was then digested and biotinylated according to the manufacturer’s instructions. A clean-up step was performed with SPRIselect beads before library preparation. DNA concentration was quantified using the Qubit Fluorometer v4.0 (Thermo Fisher Scientific) and the Qubit HS Assay Kit, following the manufacturer’s instructions.

***Hi-C library preparation and sequencing***

Biotinylated DNA constructs were fragmented using a Covaris E220 sonicator and size selected to 400–600 bp using SPRISelect beads. DNA was enriched with Arima-HiC v2 kit Enrichment beads. End repair, A-tailing, and adapter ligation were carried out with the NEBNext Ultra II DNA Library Prep Kit (New England Biolabs), following a modified protocol where library preparation occurs while DNA remains bound to the Enrichment beads. Library amplification was performed using KAPA HiFi HotStart mix and a custom Unique Dual Index (UDI) barcode set (Integrated DNA Technologies). Depending on sample concentration and biotinylation percentage determined at the crosslinking stage, libraries were amplified with 10–16 PCR cycles. Post-PCR clean-up was performed with SPRISelect beads. Libraries were quantified using the AccuClear Ultra High Sensitivity dsDNA Standards Assay Kit (Biotium) and a FLUOstar Omega plate reader (BMG Labtech).

Prior to sequencing, libraries were normalised to 10 ng/μL. Normalised libraries were quantified again to create equimolar and/or weighted 2.8 nM pools. Pool concentrations were checked using the Agilent 4200 TapeStation (Agilent) with High Sensitivity D500 reagents before sequencing. Sequencing was performed using paired-end 150 bp reads on the Illumina NovaSeq 6000.

### Genome assembly

Prior to assembly of the PacBio HiFi reads, a database of *k*-mer counts ( *k* = 31) was generated from the filtered reads using FastK. GenomeScope2 ( Ranallo-Benavidez *et al.*, 2020) was used to analyse the *k*-mer frequency distributions, providing estimates of genome size, heterozygosity, and repeat content.

The HiFi reads were assembled using Hifiasm in Hi-C phasing mode ( Cheng *et al.*, 2021, 2022), producing two haplotypes. Hi-C reads ( Rao *et al.*, 2014) were mapped to the primary contigs using bwa-mem2 ( Vasimuddin *et al.*, 2019). Contigs were further scaffolded with Hi-C data in YaHS ( Zhou *et al.*, 2023), using the --break option for handling potential misassemblies. The scaffolded assemblies were evaluated using Gfastats ( Formenti *et al.*, 2022), BUSCO ( Manni *et al.*, 2021) and MerquryFK ( Rhie *et al.*, 2020). The organelle genomes were assembled using OATK ( Zhou *et al.*, 2025).

### Assembly curation

The assembly was decontaminated using the Assembly Screen for Cobionts and Contaminants ( ASCC) pipeline. TreeVal was used to generate the flat files and maps for use in curation. Manual curation was conducted primarily in PretextView and HiGlass ( Kerpedjiev *et al.*, 2018). Scaffolds were visually inspected and corrected as described by Howe *et al*. (2021). Manual corrections included seven breaks and 19 joins. This reduced the scaffold count by 6.9%. The curation process is described at sanger-tol/curation-resources . PretextSnapshot was used to generate a Hi-C contact map of the final assembly.

### Assembly quality assessment

The MerquryFK tool ( Rhie *et al.*, 2020) was run in a Singularity container ( Kurtzer *et al.*, 2017) to evaluate *k*-mer completeness and assembly quality for both haplotypes using the *k*-mer database ( *k* = 31) computed prior to genome assembly. The analysis outputs included assembly QV scores and completeness statistics.

The genome was analysed using the BlobToolKit pipeline, a Nextflow implementation of the earlier Snakemake version ( Challis *et al.*, 2020). PacBio reads were aligned to the assembly using minimap2 ( Li, 2018) and processed with SAMtools ( Danecek *et al.*, 2021) to generate coverage tracks. The pipeline runs BUSCO ( Manni *et al.*, 2021) using lineages identified from the NCBI Taxonomy ( Schoch *et al.*, 2020). For the three basal BUSCO lineages, eukaryota, bacteria and archaea, BUSCO genes are aligned to the UniProt Reference Proteomes database ( Bateman *et al.*, 2023) using DIAMOND BLASTp ( Buchfink *et al.*, 2021). The genome assembly is divided into chunks based on the density of BUSCO genes from the closest taxonomic lineage, and each chunk is aligned to the UniProt Reference Proteomes database using DIAMOND BLASTx. Sequences without DIAMOND BLASTx hits are extracted using seqtk and aligned to the NT database using BLASTn ( Altschul *et al.*, 1990). The outputs are consolidated into a BlobDir for visualisation in BlobToolKit. The BlobToolKit pipeline was developed using nf-core tooling ( Ewels *et al.*, 2020) and MultiQC ( Ewels *et al.*, 2016), with containerisation through Docker ( Merkel, 2014) and Singularity ( Kurtzer *et al.*, 2017).

## Genome sequence report

### Sequence data

**Figure 2.**
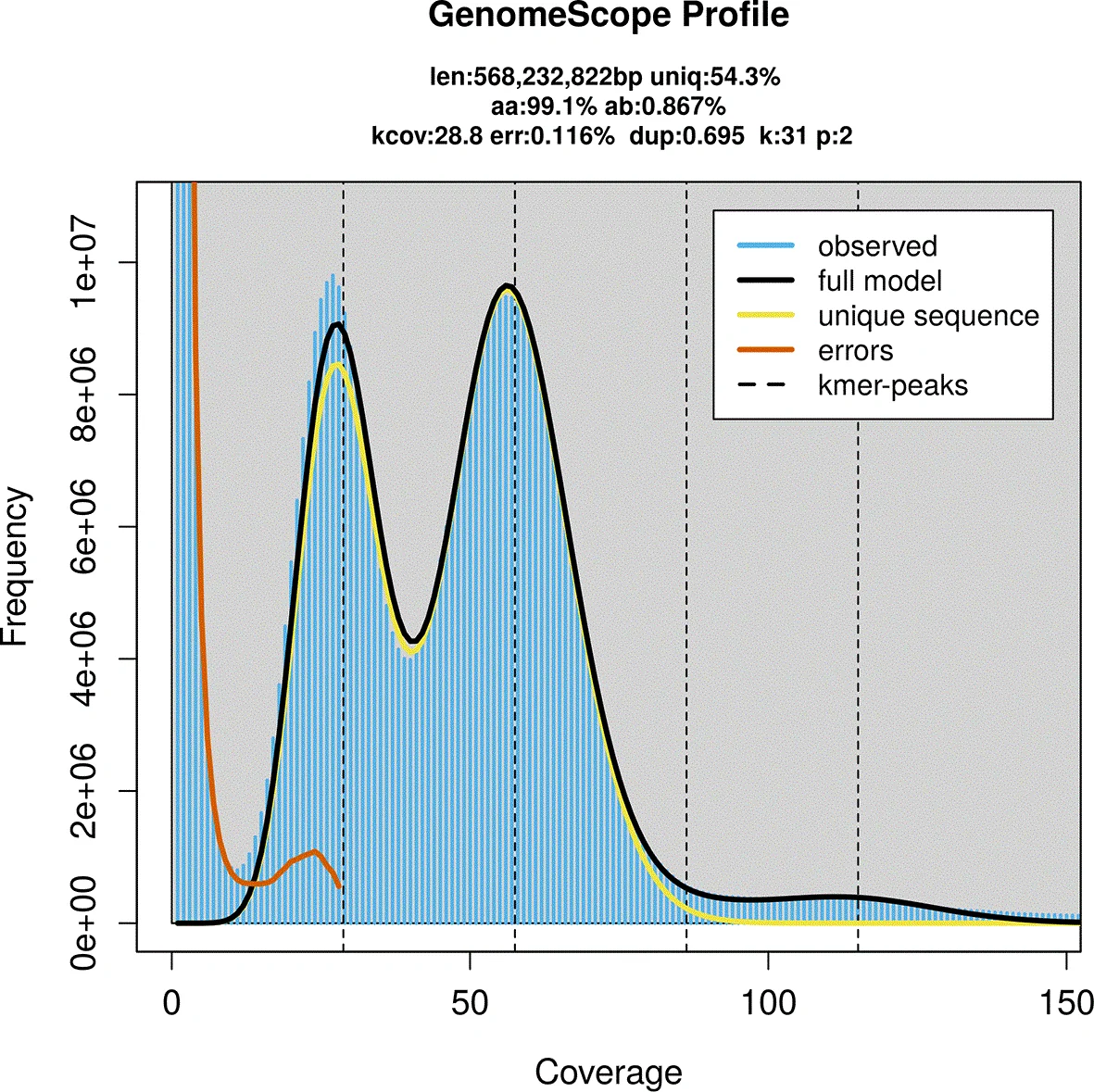
Frequency distribution of *k*-mers generated using GenomeScope2. The plot shows observed and modelled *k*-mer spectra, providing estimates of genome size, heterozygosity, and repeat content based on unassembled sequencing reads.

The genome of a specimen of *Aesculus hippocastanum* was sequenced using Pacific Biosciences single-molecule HiFi long reads, generating 33.95 Gb (gigabases) from 2.24 million reads, which were used to assemble the genome. GenomeScope2.0 analysis estimated the haploid genome size at 568.97 Mb, with a heterozygosity of 0.87% and repeat content of 45.69% ( Figure 2). Using flow cytometry, the genome size (1C-value) of the sample was estimated to be 0.64 pg, equivalent to 620.00 Mb. These estimates guided expectations for the assembly. Based on the estimated genome size, the sequencing data provided approximately 58× coverage. Hi-C sequencing produced 100.46 Gb from 332.66 million read pairs, which were used to scaffold the assembly. Table 1 summarises the specimen and sequencing details.

**Table 1.** Specimen and sequencing data for *Aesculus hippocastanum* (BioProject PRJEB85396).

Platform	PacBio HiFi	Hi-C
**ToLID**	drAesHipp1	drAesHipp1
**Specimen ID**	EDTOL01199	EDTOL01199
**BioSample (source individual)**	SAMEA10241428	SAMEA10241428
**BioSample (tissue)**	SAMEA10241542	SAMEA10241542
**Tissue**	leaf	leaf
**Instrument**	Revio	Illumina NovaSeq 6000
**Run accessions**	ERR14231596	ERR14242311
**Read count total**	2.24 million reads	332.66 million read pairs
**Base count total**	33.95 Gb	100.46 Gb

### Assembly statistics

The genome was assembled into two haplotypes using Hi-C phasing. Haplotype 1 was curated to chromosome level, while haplotype 2 was assembled to scaffold level. The final assembly has a total length of 513.59 Mb in 133 scaffolds, with 373 gaps, and a scaffold N50 of 25.74 Mb ( Table 2).

**Table 2.** Genome assembly statistics for *Aesculus hippocastanum.*

Genome assembly	Haplotype 1	Haplotype 2
**Assembly name**	drAesHipp1.hap1.1	drAesHipp1.hap2.1
**Assembly accession**	GCA_965199115.1	GCA_965198745.1
**Assembly level**	chromosome	scaffold
**Span (Mb)**	513.59	506.90
**Number of chromosomes**	20	-
**Number of contigs**	506	462
**Contig N50**	2.09 Mb	2.02 Mb
**Number of scaffolds**	133	108
**Scaffold N50**	25.74 Mb	25.94 Mb
**Longest scaffold length (Mb)**	36.06	35.18
**Organelles**	Mitochondrial genome: 507.21 kb; Plastid genome: 156.08 kb	-

The scaffold count follows the NCBI assembly statistics and excludes organelle assemblies, which are reported separately where present.

**Figure 3.**
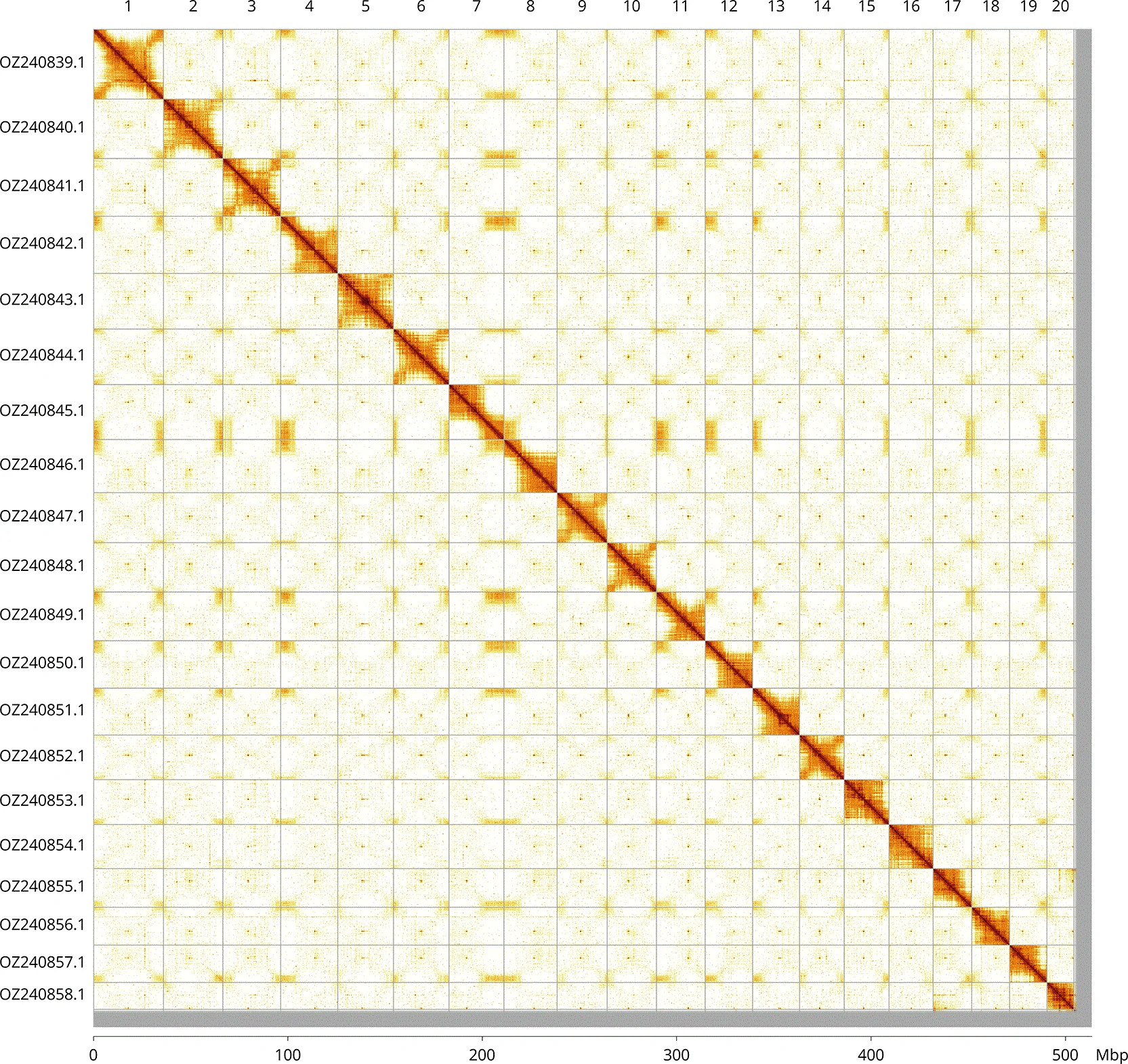
Hi-C contact map of the *Aesculus hippocastanum* genome assembly. Assembled chromosomes are shown in order of size and labelled along the axes, with a megabase scale shown below. The plot was generated using PretextSnapshot.

Most of the assembly sequence (98.22%) was assigned to 20 chromosomal-level scaffolds. These chromosome-level scaffolds, confirmed by Hi-C data, are named according to size ( Figure 3; Table 3).

**Table 3.** Chromosomal pseudomolecules in the haplotype 1 genome assembly of *Aesculus hippocastanum* drAesHipp1.

INSDC accession	Molecule	Length (Mb)	GC%
OZ240839.1	1	36.06	36.50
OZ240840.1	2	30.61	36.50
OZ240841.1	3	29.74	36.50
OZ240842.1	4	29.40	36.50
OZ240843.1	5	28.63	37.50
OZ240844.1	6	28.48	36
OZ240845.1	7	28.37	36.50
OZ240846.1	8	27.32	37
OZ240847.1	9	25.74	36
OZ240848.1	10	25.30	36.50
OZ240849.1	11	25.10	36
OZ240850.1	12	24.47	36.50
OZ240851.1	13	24.07	36.50
OZ240852.1	14	23.04	36
OZ240853.1	15	23.01	36.50
OZ240854.1	16	22.73	36.50
OZ240855.1	17	19.86	37
OZ240856.1	18	19.47	37
OZ240857.1	19	19.20	36.50
OZ240858.1	20	13.86	36.50

The mitochondrial genome (length 507.21 kb, OZ240859.1) and plastid genome (length 156.08 kb, OZ240860.1) were also assembled. These sequences are included as contigs in the multifasta file of the genome submission and as standalone records.

### Assembly quality metrics

**Figure 4.**
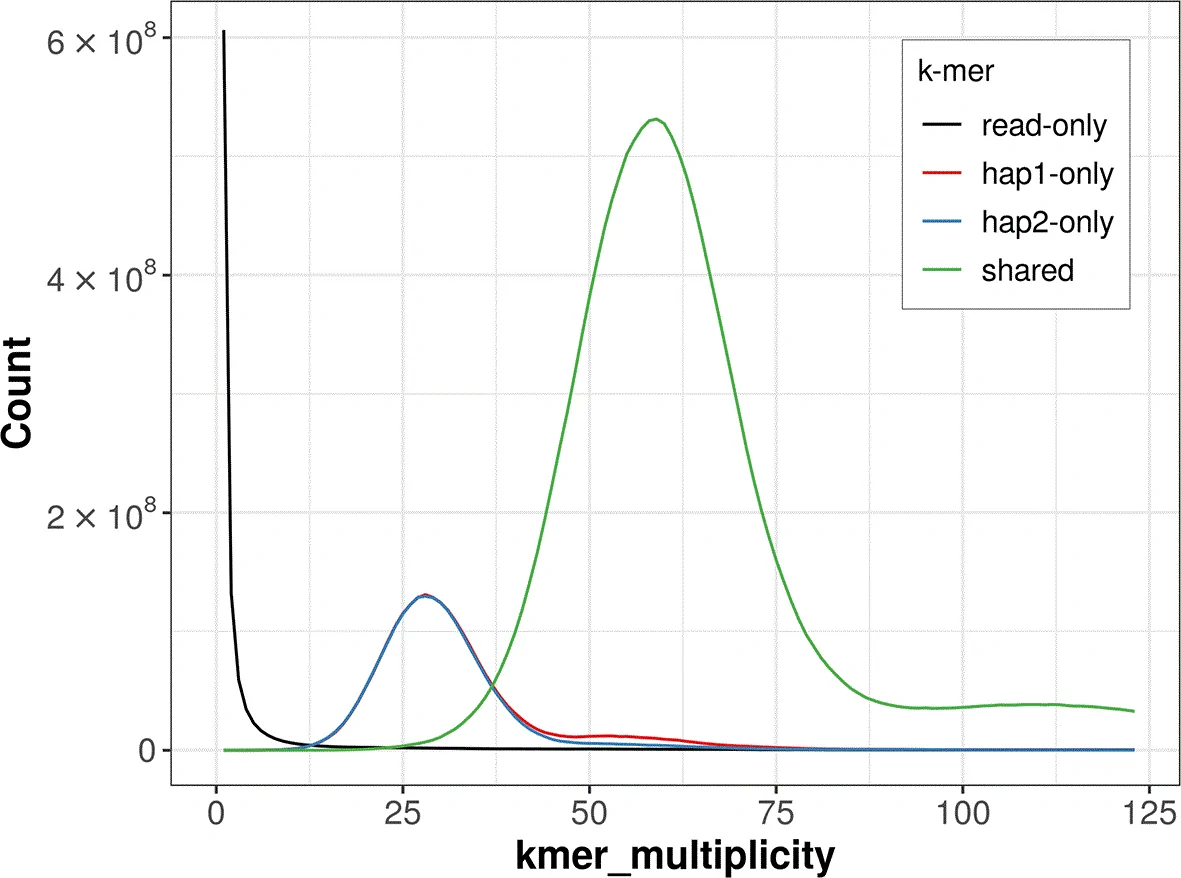
Evaluation of *k*-mer completeness using MerquryFK. This plot illustrates the recovery of *k*-mers from the original read data in the final assemblies. The horizontal axis represents *k*-mer multiplicity, and the vertical axis shows the number of *k*-mers. The black curve represents *k*-mers that appear in the reads but are not assembled. The green curve corresponds to *k*-mers shared by both haplotypes, and the red and blue curves show *k*-mers found only in one of the haplotypes.

For haplotype 1, the estimated QV is 64.4, and for haplotype 2, 65.0. When the two haplotypes are combined, the assembly achieves an estimated QV of 64.7. The *k*-mer completeness is 81.23% for haplotype 1, 80.48% for haplotype 2, and 98.99% for the combined haplotypes ( Figure 4).

**Figure 5.**
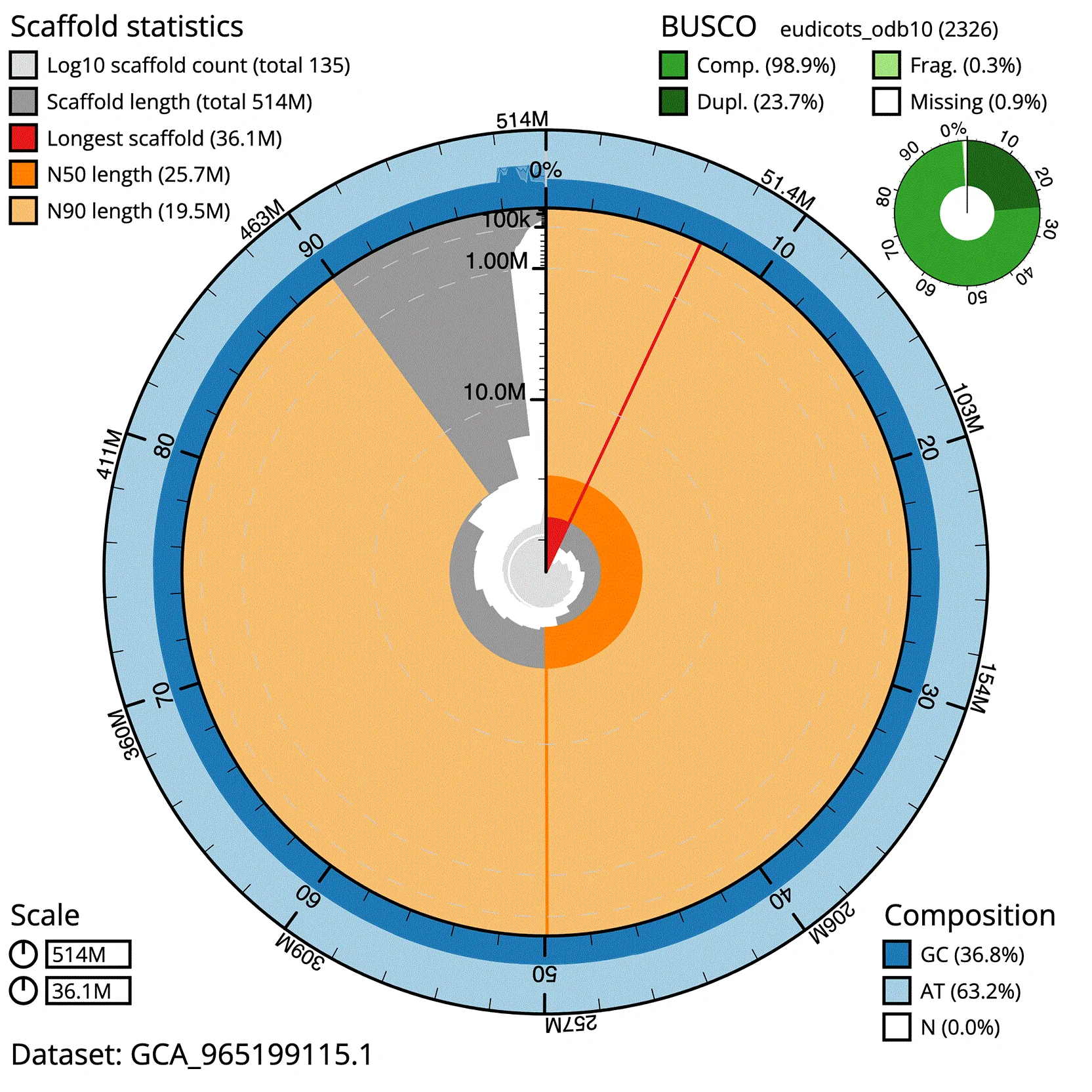
Assembly metrics for drAesHipp1.hap1.1. The BlobToolKit snail plot provides an overview of assembly metrics and BUSCO gene completeness. The circumference represents the length of the whole genome sequence, and the main plot is divided into 1 000 bins around the circumference. The outermost blue tracks display the distribution of GC, AT, and N percentages across the bins. Scaffolds are arranged clockwise from longest to shortest and are depicted in dark grey. The longest scaffold is indicated by the red arc, and the deeper orange and pale orange arcs represent the N50 and N90 lengths. A light grey spiral at the centre shows the cumulative scaffold count on a logarithmic scale. A summary of complete, fragmented, duplicated, and missing BUSCO genes in the set is presented at the top right. An interactive version of this figure can be accessed on the BlobToolKit viewer.

**Figure 6.**
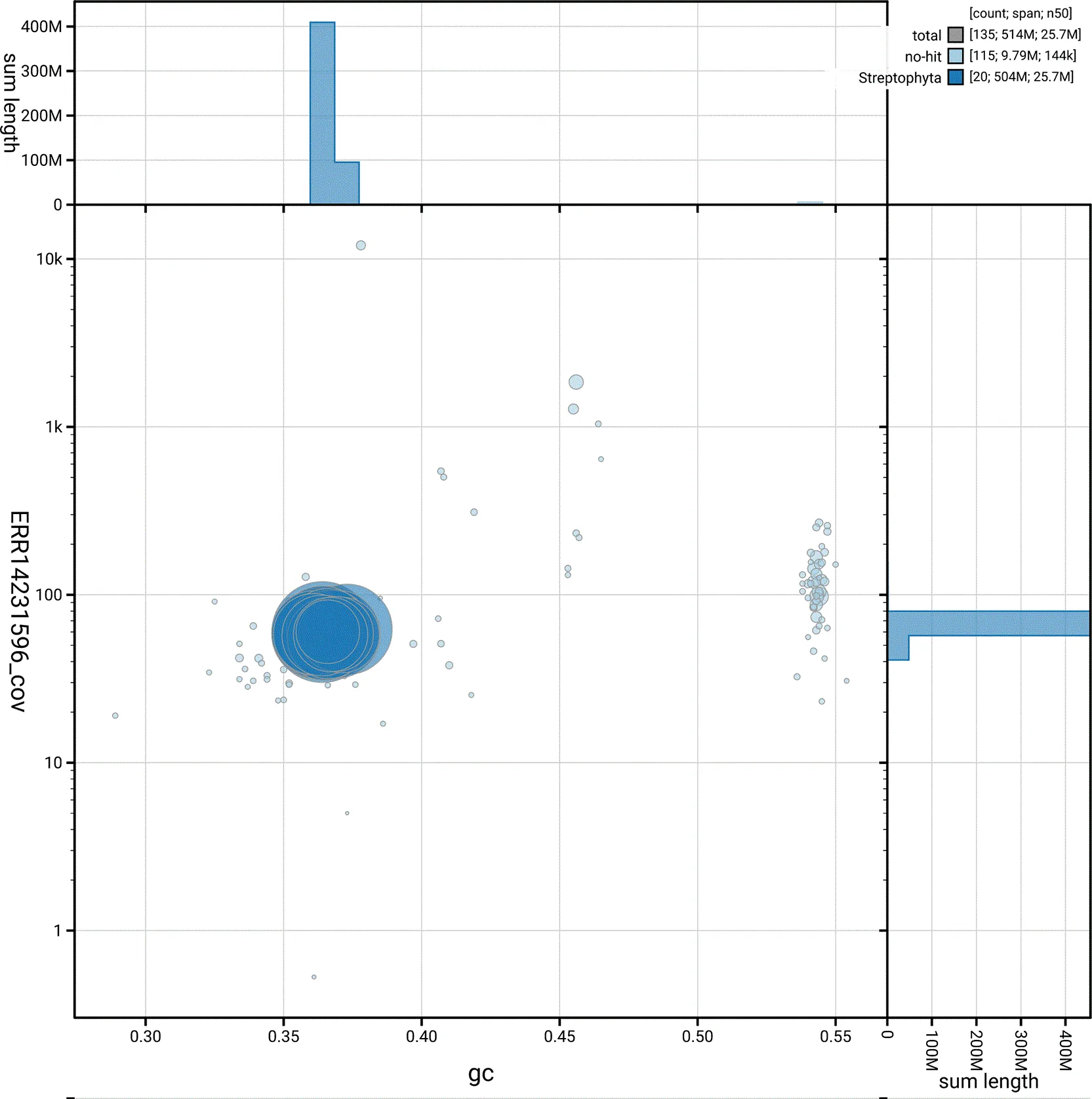
BlobToolKit blob plot for drAesHipp1.hap1.1. The plot shows base coverage (vertical axis) and GC content (horizontal axis). The circles represent scaffolds, with the size proportional to scaffold length and the colour representing phylum membership. The histograms along the axes display the total length of sequences distributed across different levels of coverage and GC content. An interactive version of this figure is available on the BlobToolKit viewer.

BUSCO analysis using the eudicots_odb10 reference set ( *n* = 2 326) identified 98.9% of the expected gene set (single = 75.2%, duplicated = 23.7%) for haplotype 1. The snail plot in Figure 5 summarises the scaffold length distribution and other assembly statistics for haplotype 1. The blob plot in Figure 6 shows the distribution of scaffolds by GC proportion and coverage for haplotype 1.

Table 4 lists the assembly metric benchmarks adapted from Rhie *et al.* (2021) and the Earth BioGenome Project Report on Assembly Standards January 2026. The EBP metric calculated for haplotype 1 is **6.C.Q64**, meeting the recommended 6.C.Q40 reference standard.

**Table 4.** Earth Biogenome Project summary metrics for the *Aesculus hippocastanum* assembly.

Measure	Value	Benchmark
EBP summary (haplotype 1)	6.C.Q64	6.C.Q40
Contig N50 length	2.09 Mb	≥ 1 Mb
Scaffold N50 length	25.74 Mb	= chromosome N50
Consensus quality (QV)	Haplotype 1: 64.4; haplotype 2: 65.0; combined: 64.7	≥ 40
*k*-mer completeness	Haplotype 1: 81.23%; Haplotype 2: 80.48%; combined: 98.99%	≥ 90%
BUSCO	Haplotype 1: C:98.9% [S:75.2%, D:23.7%], F:0.3%, M:0.9%, n:2 326; Haplotype 2: C:98.3% [S:75.2%, D:23.1%], F:0.3%, M:1.4%, n:2 326	S > 90%; D < 5%
Percentage of assembly assigned to chromosomes	98.22%	≥ 90%

**Notes:** The EBP summary uses log10(Contig N50); chromosome-level (C) or log10(Scaffold N50); Q (Merqury QV). BUSCO: C = complete; S = single-copy; D = duplicated; F = fragmented; M = missing; n = orthologues

## Author information

• Members of the Royal Botanic Garden Edinburgh Genome Acquisition Lab
• Members of the Plant Genome Sizing Collective
• Members of the Darwin Tree of Life Barcoding collective
• Members of the Wellcome Sanger Institute Tree of Life Management, Samples and Laboratory team
• Members of Wellcome Sanger Institute Scientific Operations – Sequencing Operations
• Members of the Wellcome Sanger Institute Tree of Life Core Informatics team
• Members of the Tree of Life Core Informatics collective
• Members of the Darwin Tree of Life Consortium

## Wellcome sanger institute – Legal and governance

The materials that have contributed to this genome note have been supplied by a Darwin Tree of Life Partner. The submission of materials by a Darwin Tree of Life Partner is subject to the **‘Darwin Tree of Life Project Sampling Code of Practice’**, which can be found in full on the Darwin Tree of Life website. By agreeing with and signing up to the Sampling Code of Practice, the Darwin Tree of Life Partner agrees they will meet the legal and ethical requirements and standards set out within this document in respect of all samples acquired for, and supplied to, the Darwin Tree of Life Project. Further, the Wellcome Sanger Institute employs a process whereby due diligence is carried out proportionate to the nature of the materials themselves, and the circumstances under which they have been/are to be collected and provided for use. The purpose of this is to address and mitigate any potential legal and/or ethical implications of receipt and use of the materials as part of the research project, and to ensure that in doing so we align with best practice wherever possible. The overarching areas of consideration are:

• Ethical review of provenance and sourcing of the material
• Legality of collection, transfer and use (national and international)

Each transfer of samples is further undertaken according to a Research Collaboration Agreement or Material Transfer Agreement entered into by the Darwin Tree of Life Partner, Genome Research Limited (operating as the Wellcome Sanger Institute), and in some circumstances, other Darwin Tree of Life collaborators.

## Data Availability

European Nucleotide Archive: Aesculus hippocastanum (common horse chestnut). Accession number PRJEB85396; https://identifiers.org/ena.embl/PRJEB85396. The genome sequence is released openly for reuse. The *Aesculus hippocastanum* genome sequencing initiative is part of the Darwin Tree of Life Project (PRJEB40665) and the Sanger Institute Tree of Life Programme (PRJEB43745). All raw sequence data and the assembly have been deposited in INSDC databases. The genome will be annotated using available RNA-Seq data and presented through the Ensembl pipeline at the European Bioinformatics Institute. Raw data and assembly accession identifiers are reported in Tables 1 and 2. Pipelines used for genome assembly at the WSI Tree of Life are available at https://pipelines.tol.sanger.ac.uk/pipelines. Table 5 lists software versions used in this study.
