# Supplementary material for: The genome sequence of Aesculus hippocastanum L., 1753 (Sapindales: Sapindaceae)
Source: Wellcome Open Res. 2026 Aug 31;11:563. [Version 1] doi: 10.12688/wellcomeopenres.27204.1 (PMC13594604; doi:10.12688/wellcomeopenres.27204.1)
Supplement: Peer review history

# Reviewer report: Reviewer response for version 1

**DOI:** 10.21956/wellcomeopenres.29944.r166714

**Published:** 2026-10-01

**Authors:** Chaehee Lee

**Affiliations:** 1 University of California Davis, Davis, California, USA

In this data note, Ruhsam and colleagues present a haplotype-resolved genome assembly of *Aesculus hippocastanum* (drAesHipp1). Haplotype 1 was curated to chromosome level in size of 513.59 Mb, with 98.22% placed in 20 pseudomolecules, consistent with 2n = 40. Haplotype 2 (506.90 Mb) is released at scaffold level. They also provided mitochondrial and plastid genomes. Overall, this is a high-quality, useful resource, and I support approval. I have a few points that I think should be addressed, followed by minor corrections.

 Comment:

 - BUSCO duplication and the EBP benchmarks. Table 4 lists S > 90% and D < 5% as benchmarks, but the assembly has S = 75.2% and D = 23.7% (haplotype 1) and 23.1% (haplotype 2). The text states that the assembly meets the 6.C.Q40 standard but doesn't say that the BUSCO benchmark is not met. Since 98.22% of haplotype 1 is on chromosomes and only about 9 Mb is unplaced, most duplicated BUSCOs are probably on different chromosomes rather than retained haplotigs. That would point to a genuine duplication in the lineage, not an assembly artifact. It would be helpful if the authors report how many duplicated BUSCO pairs fall on different chromosomes versus on unplaced scaffolds, and add one or two sentences on a possible whole-genome duplication. Comparing with the published *A. chinensis* or *A. wilsonii* assemblies would be the easiest way to check whether this duplication is shared wihtin the genus.

 - Haplotype 2 has a scaffold N50 of 25.94 Mb, so most of it appears to be in chromosome-scale scaffolds. Reporting how much of hap2 falls in its 20 largest scaffolds would be really helpful. The simple way could be a dot plot between hap1 and hap2.

 Please also clarify:

 - Whether YaHS was run on both haplotypes (the text says Hi-C reads were mapped to the "primary contigs," which is unclear in Hi-C phasing mode)

 - How much sequence ASCC removed as contamination, if any

 Minor corrections:

 - In Nucleic acid extraction, “65.000 mg” should be “65 mg”

 - Coverage is given as "approximately 58" based on "the estimated genome size." 33.95 Gb gives about 60x against the GenomeScope estimate and about 55x against flow cytometry, so please state which estimate was used. The multiplication sign also appears to be missing.

Are sufficient details of methods and materials provided to allow replication by others?

Yes

Is the rationale for creating the dataset(s) clearly described?

Yes

Are the datasets clearly presented in a useable and accessible format?

Yes

Are the protocols appropriate and is the work technically sound?

Yes

Reviewer Expertise:

Plant genomics

I confirm that I have read this submission and believe that I have an appropriate level of expertise to confirm that it is of an acceptable scientific standard, however I have significant reservations, as outlined above.

# Reviewer report: Reviewer response for version 1

**DOI:** 10.21956/wellcomeopenres.29944.r166717

**Published:** 2026-09-21

**Authors:** Marco Todesco

**Affiliations:** 1 Michael Smith Laboratories, University of British Columbia, Vancouver, Canada

This manuscript describes the generation of a genome assembly for horse chestnut ( *Aesculus hippocastanum*), as part of the Darwin Tree of Life (DToL) project.

 I was very excited to read this data note, as horse chestnuts are some of my favourite plants, and I was not disappointed. The genome assembly is of very high quality, and the manuscript provides all necessary information about its generation, as well as interesting background information on the species.

 The vast majority (>98%) of the assembly is assigned to the 20 expected chromosomes. Duplication is a bit high in both haplotypes (~23% BUSCO duplicates), despite the fact that they are quite a bit smaller than the expected size ( ~510 Mbp vs the expected 570-620 Mbp based on k-mer counts and flow cytometry) - it would be interesting to have a short discussion on why that might be the case. The Hi-C heatmaps are very clean.

 There are just a couple of minor corrections to the text that it would be useful to make:

 - At the end of the "nucleic acid extraction" methods section, the total DNA yield post-fragmentation is missing ("...and a yield of ng"

 - In the second sentence of the "assembly statistics" section, it would be useful to clarify that the reported values are for haplotype 1 (only that haplotype was scaffolded, so it might be obvious, but it took me a second to realize that)

Are sufficient details of methods and materials provided to allow replication by others?

Yes

Is the rationale for creating the dataset(s) clearly described?

Yes

Are the datasets clearly presented in a useable and accessible format?

Yes

Are the protocols appropriate and is the work technically sound?

Yes

Reviewer Expertise:

Plant genetics, genomics, plant adaptation

I confirm that I have read this submission and believe that I have an appropriate level of expertise to confirm that it is of an acceptable scientific standard.

# Reviewer report: Reviewer response for version 1

**DOI:** 10.21956/wellcomeopenres.29944.r166716

**Published:** 2026-09-16

**Authors:** June Labbancz

**Affiliations:** 1 Texas A&M University, College Station, Texas, USA

This data note describes a genomic assembly for an *Aesculus hippocastanum* individual sampled in the United Kingdom. A botanical description of the species and its ecological significance are outlined quite clearly in the background section. The methods described are reasonable for the generation of a genomic assembly and utilize standardized procedures. Finally, summary statistics describing the assembly are provided. The statistics and figures provided indicate a genomic assembly of high quality, with a particularly high degree of completeness (98.9%) and correctness (QV 64.7) in the assembly as assessed by BUSCO and MerquryFK, respectively. Contiguity is acceptable with a contig N50 of 2.09 Mb. Organelle genome assemblies are also present. The generated assembly is easily accessible from the publication, facilitating reuse.

 I have no major concerns about the assembly or the data note. In the background section, briefly citing and discussing existing genomic assemblies within *Aesculus,* such as *Aesculus wilsonii *and *Aesculus chinensis*, could help provide greater scientific context for this work.

Are sufficient details of methods and materials provided to allow replication by others?

Yes

Is the rationale for creating the dataset(s) clearly described?

Yes

Are the datasets clearly presented in a useable and accessible format?

Yes

Are the protocols appropriate and is the work technically sound?

Yes

Reviewer Expertise:

Plant Genomics

I confirm that I have read this submission and believe that I have an appropriate level of expertise to confirm that it is of an acceptable scientific standard.
